# Usability, Engagement, and Report Usefulness of Chatbot-Based Family Health History Data Collection: Mixed Methods Analysis

**DOI:** 10.2196/55164

**Published:** 2024-09-30

**Authors:** Michelle Hoang Nguyen, João Sedoc, Casey Overby Taylor

**Affiliations:** 1 Institute for Computational Medicine Johns Hopkins University Baltimore, MD United States; 2 Department of Biomedical Engineering Johns Hopkins University School of Medicine Baltimore, MD United States; 3 Department of Technology, Operations and Statistics Stern School of Business New York University New York, NY United States; 4 Division of General Internal Medicine Department of Medicine Johns Hopkins University School of Medicine Baltimore, MD United States

**Keywords:** family health history, chatbots, conversational agents, digital health tools, usability, engagement, report usefulness, evaluation, crowdsourcing, mixed methods

## Abstract

**Background:**

Family health history (FHx) is an important predictor of a person’s genetic risk but is not collected by many adults in the United States.

**Objective:**

This study aims to test and compare the usability, engagement, and report usefulness of 2 web-based methods to collect FHx.

**Methods:**

This mixed methods study compared FHx data collection using a flow-based chatbot (KIT; the curious interactive test) and a form-based method. KIT’s design was optimized to reduce user burden. We recruited and randomized individuals from 2 crowdsourced platforms to 1 of the 2 FHx methods. All participants were asked to complete a questionnaire to assess the method’s usability, the usefulness of a report summarizing their experience, user-desired chatbot enhancements, and general user experience. Engagement was studied using log data collected by the methods. We used qualitative findings from analyzing free-text comments to supplement the primary quantitative results.

**Results:**

Participants randomized to KIT reported higher usability than those randomized to the form, with a mean System Usability Scale score of 80.2 versus 61.9 (*P*<.001), respectively. The engagement analysis reflected design differences in the onboarding process. KIT users spent less time entering FHx information and reported more conditions than form users (mean 5.90 vs 7.97 min; *P*=.04; and mean 7.8 vs 10.1 conditions; *P*=.04). Both KIT and form users somewhat agreed that the report was useful (Likert scale ratings of 4.08 and 4.29, respectively). Among desired enhancements, personalization was the highest-rated feature (188/205, 91.7% rated medium- to high-priority). Qualitative analyses revealed positive and negative characteristics of both KIT and the form-based method. Among respondents randomized to KIT, most indicated it was easy to use and navigate and that they could respond to and understand user prompts. Negative comments addressed KIT’s personality, conversational pace, and ability to manage errors. For KIT and form respondents, qualitative results revealed common themes, including a desire for more information about conditions and a mutual appreciation for the multiple-choice button response format. Respondents also said they wanted to report health information beyond KIT’s prompts (eg, personal health history) and for KIT to provide more personalized responses.

**Conclusions:**

We showed that KIT provided a usable way to collect FHx. We also identified design considerations to improve chatbot-based FHx data collection: First, the final report summarizing the FHx collection experience should be enhanced to provide more value for patients. Second, the onboarding chatbot prompt may impact data quality and should be carefully considered. Finally, we highlighted several areas that could be improved by moving from a flow-based chatbot to a large language model implementation strategy.

## Introduction

### Background

Comprehensive and high-quality family health history (FHx) is a valuable tool for research and patient care, even as the genomic medicine landscape evolves. As next-generation sequencing costs decline, genotype-first approaches are being explored as an alternative to traditional phenotypic ascertainment or clinical informatics approaches. Even in this era of genomic advancement, FHx remains a valuable risk predictor, especially for complex conditions not fully explained by genomic factors alone [1,2]. For example, in a population genetics screening study by Bylstra et al [3], participants with higher cancer risk based on FHx assessment had a higher prevalence of clinically actionable variants in American College of Medical Genetics secondary findings (version 2.0) cancer genes than both participants with average FHx-derived cancer risk and those who had no documented FHx.

Although FHx is widely recognized as a crucial step in genetic risk assessment, less than 50% of Americans report actively collecting their FHx [4]. Furthermore, most patients do not have complete FHx in their electronic health record (EHR) [5].

Barriers to collecting FHx have persisted for both patients and providers. Providers face challenges in collecting FHx due to a lack of time during patient visits and limitations of current tools to document FHx comprehensively [6-8]. Patient challenges have included a lack of knowledge of the value of FHx to assess disease risk, the great effort required to confirm conditions present among family members, the need to regularly update FHx to maintain accuracy, and insufficient encouragement from clinicians to complete FHx documentation [4,6,9,10]. Innovative digital health strategies may help to address patient and provider challenges by providing mechanisms to reduce clinician burden and improve FHx completion rate by empowering patients to electronically complete their FHx at a time and place that is convenient for them. Furthermore, for patients, digital health strategies can facilitate access to relevant educational materials and mechanisms to save and update FHx over time.

In recent years, there have been increasing efforts to advance FHx collection with the development, implementation, and use of web-based tools [11-14]. Many of the digital health strategies to date are patient-facing and involve patient-entered information. These strategies are promising. For example, in the Cancer Health Assessments Reaching Many study, Mittendorf et al [15] adapted a web-based survey application for Lynch syndrome risk assessment from a provider-facing to a patient-facing format that supported computer and mobile use. Adaptations were designed to be patient-friendly by presenting survey questions about family history one relative at a time (or a small group of relatives at a time), including literacy aids, and to computationally determine side of the family instead of relying on the patient. In another study designed to assess the effectiveness of a cancer genetic service delivery among a diverse patient population, Mittendorf et al [16] found that patients preferred FHx self-assessment and data entry using an electronic history tool over clinician-gathered history. Factors favorable among study participants included being self-paced, private, convenient, and allowing them to gather more detailed information from relatives without time constraints [16].

While electronic tools may have their benefits, a subset of individuals from the Cancer Health Assessments Reaching Many study who preferred clinician-gathered history noted that clinicians could provide more tailored responses and educational information [16]. Those findings suggest a need to support tailoring and information retrieval features that may be difficult to implement using standard form-based methods.

A growing number of studies explore more advanced interfaces, such as conversational agents for health data collection and risk assessment, that may support features standard form-based methods cannot [17-25]. For example, Soni et al [26] compared the usability of a health data collection chatbot, Dokbot, against a standard REDCap (Research Electronic Data Capture; Vanderbilt University) form. Dokbot is a Health Insurance Portability and Accountability Act–compliant application accessible on both desktop and mobile devices that allows users to complete health data collection by chatting with a customizable chatbot avatar through multiple-choice button responses [27]. Soni et al [26] found that a collection chatbot is more engaging and interactive than form-based methods but does not appear more usable than a traditional web-based form [26]. However, more work is needed to understand chatbot-specific usability, the impact of chatbot-specific features on usability, and to determine what features users would prioritize for future development.

### Objectives

To address the challenges of collecting FHx and better understand the benefits and drawbacks between different web-based modalities, this paper studied the potential for a flow-based chatbot approach to collect FHx. The primary objective of this study was to understand the usability of a chatbot compared to a form-based method and observe any chatbot-specific user interface characteristics that may explain this usability. To do this, we created an interactive web-embedded chatbot to administer a 3-generational family history survey (KIT, the curious interactive test). We studied its overall usability, perceived usefulness in collecting FHx, and how engaging it was. This research is motivated by recommendations from prior work to improve the effectiveness of FHx tools and address gaps surrounding patient’s FHx knowledge [4,6]. New web-based methods, such as chatbots for FHx collection, hold promise to address these issues.

To address our research objective, we developed KIT, the FHx data collection chatbot, and a baseline form-based FHx data collection method. We compared usability measures, engagement with FHx data collection methods, and FHx summary report usefulness across these 2 methods.

## Methods

### Intervention Design

Both FHx data collection tools were developed by adapting the National Institutes of Health *All of Us* Research Program Family Health History Questionnaire, which is currently being administered on the web to *All of Us* research participants [28]. For both FHx tools, we enabled users to learn more about health conditions they might not have been familiar with by providing options to access National Institutes of Health National Library of Medicine (NLM) consumer–focused definitions from MedlinePlus [29].

**Figure 1:**
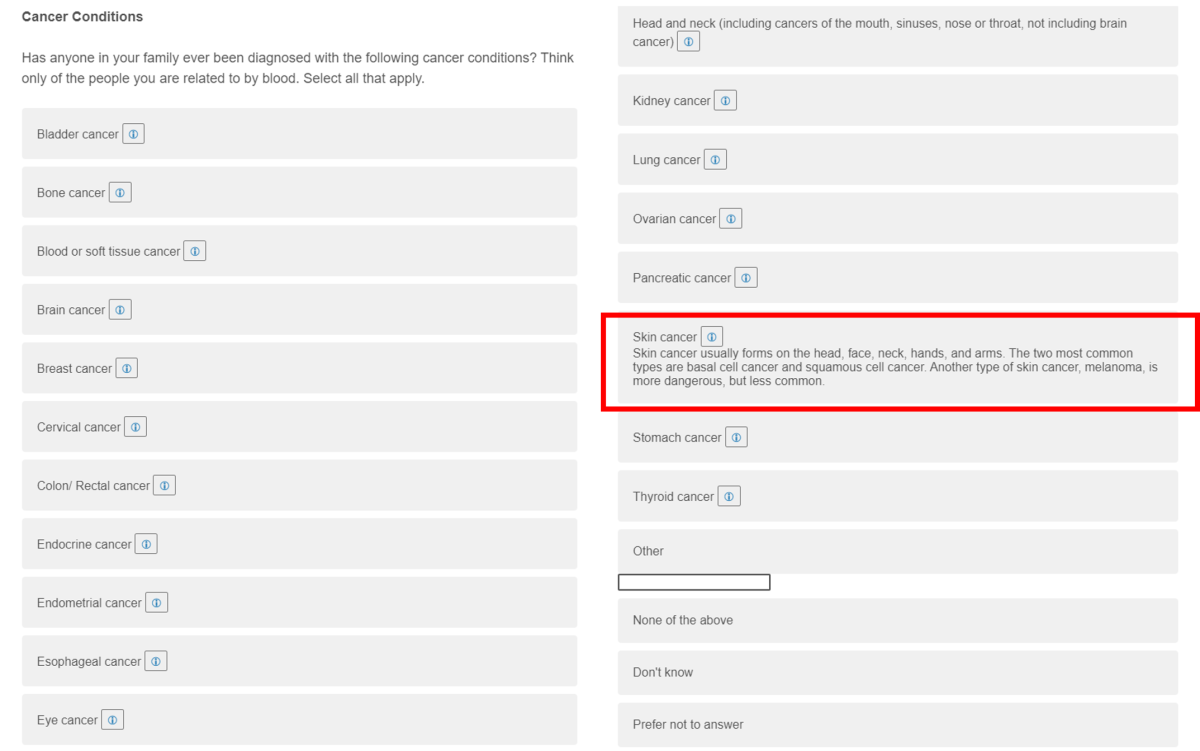
Form-based family health history tool user interface. Condition definition displayed in the red box.

Study participants were randomized to 1 of 2 FHx data collection interventions: a form-based and a chatbot-based intervention. The form-based FHx data collection method was hosted on JHMI Qualtrics. The question structure and presentation of the form remained true to the *All of Us* survey, aside from adding an information button for health conditions (Figure 1). Participants were asked to complete data entry for all 11 condition categories. A diagram is provided in Figure S1A in Multimedia Appendix 1. Data entry for FHx collection consisted of multiple-answer and multiple-choice questions. There were no free-response questions. At the end of data collection, participants were shown a copy of their responses and invited to download it to serve as a report of their FHx. The responses were in the same visual format as the survey and were available as a PDF file. More details about the form intervention questionnaire format and questions can be found in Figure S2A in Multimedia Appendix 1.

**Figure 2:**
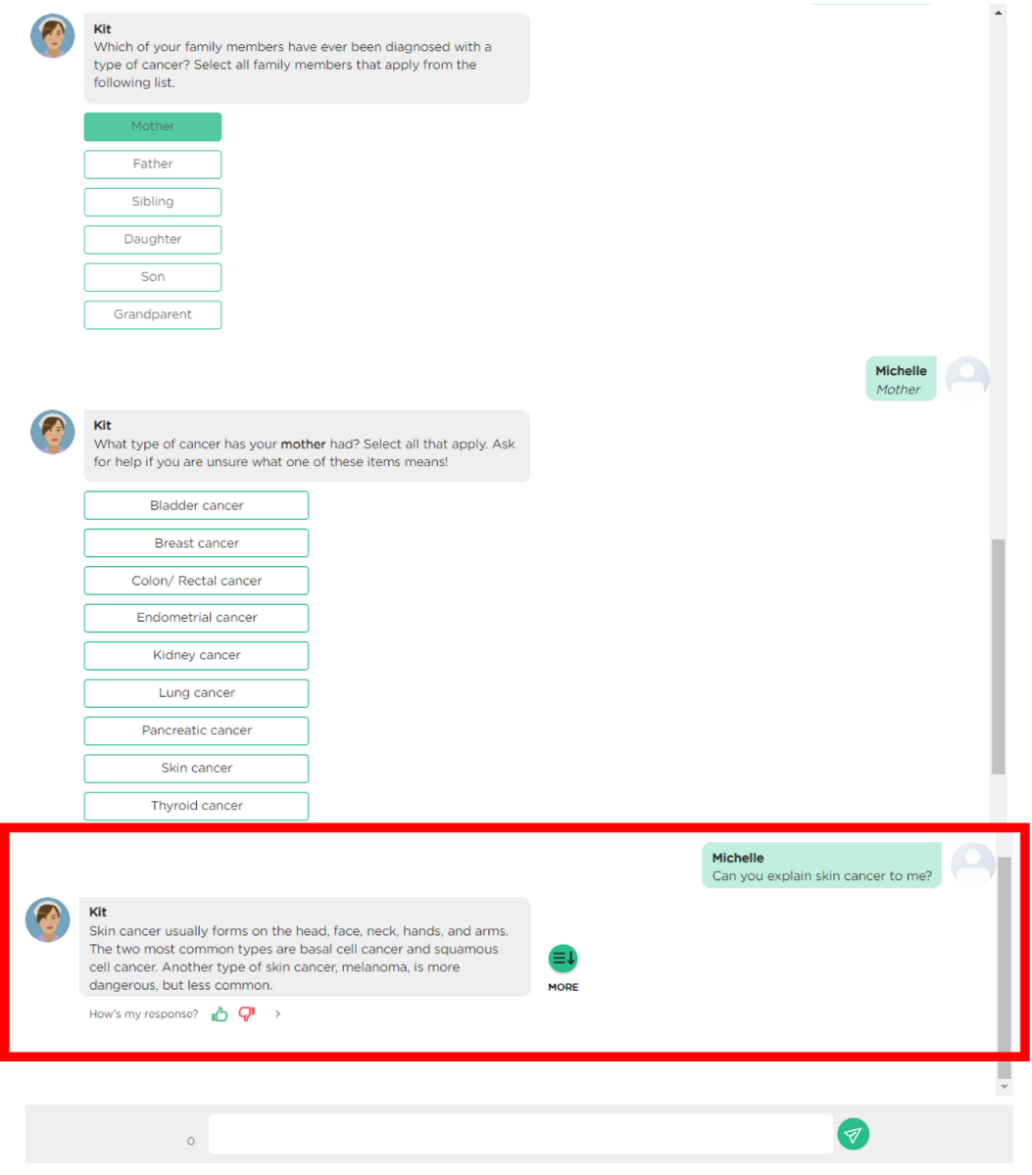
Curious interactive test (KIT), family health history chatbot, user interface on Juji. Condition question and response from KIT with KIT Avatar in the red box.

The chatbot intervention, KIT, was developed on Juji (Juji, Inc) [30]. Juji is a low-code chatbot implementation platform that reduces the technical barrier to creating chatbots. Chatbots created with Juji can be instantiated with a chatbot avatar selected from a library of available default avatars, such as the nurse figure selected for KIT. Although Juji does allow customization for more specialized chatbot behavior, such as media responses, we limited customization only when functionally necessary to move the data collection conversation forward without halting the user. Such customization included a custom topic called “Asking a question about medical information” to prevent the Question and Answer knowledge bank (which contains all the NLM MedlinePlus condition definitions) from being triggered at inappropriate instances and to allow users to input information without being incorrectly rerouted in their conversation (Figure 2). Participants were asked to select condition categories for which they knew family history among the 11 condition categories. Then, they completed data entry for the condition categories they preselected. A diagram is provided in Figure S3A in Multimedia Appendix 1 with a sample interaction video (Video S5A in Multimedia Appendix 1). The primary data entry for KIT FHx collection consisted of multiple-answer, multiple-choice questions. Users had the option to message KIT (free-response text entry) to respond (in lieu of clicking the multiple-choice button) if they had a question or if they selected “other” at any point during data collection. When users selected “other,” KIT asked the user for additional details about their data entry (eg, “Can you describe what kind of hormone/endocrine condition your father has had?”). At the end of the interaction, all participants were invited to download a copy of their conversation to serve as a report of their FHx. The conversation is a transcript in a text file format. More details about the KIT intervention format and questions appear in Multimedia Appendix 1.

The intervention designs described above reflect the improvements made after initial pilot testing of the FHx data collection tools with crowdsourced participants. From this pilot study, we tested the crowdsourced recruitment workflow to ensure acceptable response data quality and monitored user comments to improve the FHx interventions. From the comments, we reduced the latent time between KIT’s responses and implemented the FHx intervention format described earlier and in Figure S3A in Multimedia Appendix 1. More details about procedures and modifications from the pilot study are available in Multimedia Appendix 2.

### Study Design and Population

This mixed methods study compared 2 modalities to collect FHx: chatbot-based and form-based tools. All study participants completed their FHx before completing a web-based survey via Qualtrics (Qualtrics). The survey gathered both quantitative and qualitative data. Qualitative results were collected and analyzed to validate and expand on the quantitative findings related to the primary outcomes studied, that is, usability, usefulness, and engagement. A between-subjects approach was chosen to reduce participant fatigue and potential learning effects. Our approach was built on the findings of a 25-subject pilot. We drew several lessons from the pilot and applied them in this study. In summary, we improved our recruitment strategy, survey workflow, and FHx intervention. Our findings also helped us to better estimate the sample size for this study.

Eligibility for this study was limited to individuals in the United States aged >18 years who knew at least 2 first-degree relatives with at least 1 condition each. We recruited from 2 separate nonclinical study source populations, 1 from Amazon Mechanical Turk (MTurk; Amazon Inc) and 1 from Qualtrics Panels. Amazon MTurk is a web-based crowdsourcing marketplace in which businesses and researchers post tasks for registered MTurk workers to complete. Qualtrics Panels is a market research panel platform managed by Qualtrics that recruits participants to complete surveys.

Qualtrics Panel participants were recruited from various web-based sources, including website intercept recruitment, member referrals, targeted email lists, gaming sites, customer loyalty web portals, permission-based networks, and social media. Crowdsourcing has been a growing source of participant recruitment for health studies [31-33]. However, previous work has demonstrated mixed data quality when recruiting crowdsourced participants for behavioral studies [30,31]. Because of this, we aimed to recruit participants from 2 separate crowdsourced populations to validate findings and increase confidence in survey results.

The MTurk source population consisted of 580 MTurk workers who had demonstrated high-quality data for past behavioral studies. We created an MTurk Human Intelligence Task and released this incrementally, allowing 9 workers to complete the study per batch. Once deemed eligible, workers who passed the screening criteria could view the consent form. We programmed randomization through Qualtrics to assign participants to equal-sized groups at an equal rate, with a quota to keep track of assignments to either of the FHx data collection tools. We randomized to reduce intervention selection bias and ensure adequate participant counts in both arms for a fully powered study. We recruited Qualtrics Panel participants with demographic quotas approximating the US population as limited by Qualtrics Panel project managers.

**Figure 3:**
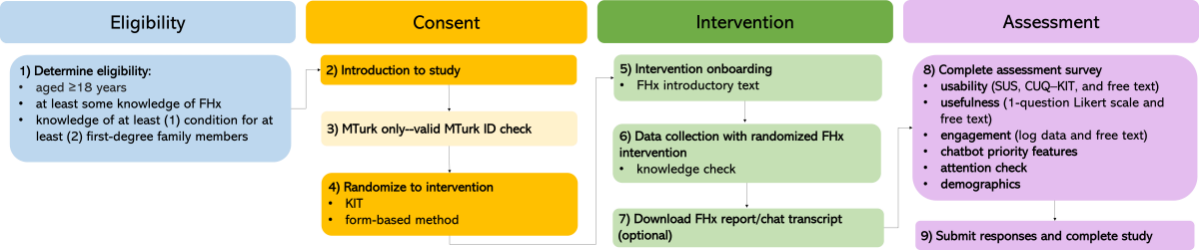
Study design flow diagram consisting of stages: eligibility, consent, intervention, and assessment. CUQ: Chatbot Usability Questionnaire; FHx: family health history; KIT: curious interactive test; MTurk: Mechanical Turk; SUS: System Usability Scale.

All recruited participants followed similar study processes (Figure 3). After determining their eligibility, we showed potential study participants contextual introductory information about genetics and family history. This was followed by a knowledge question, “Which of the following family members are not first-degree relatives?” (choices: father, sister, brother, niece) to ensure participants had thoroughly read the introductory information. If participants correctly answered “niece,” they were shown the rest of the FHx intervention. They were then redirected to proceed to the consent form. One difference between the MTurk and Qualtrics Panels procedures was that MTurk workers needed to verify a valid MTurk Worker ID before being randomized to one of the 2 FHx data collection tools. In contrast, Qualtrics Panel participants were randomized upon accepting the study invitation. After data collection, participants could download the report or chat transcript and were redirected to complete the assessment survey (Multimedia Appendix 3). Within the assessment survey, there was one attention check. If users did not correctly respond to the question, they were redirected and could not complete the survey.

### Ethical Considerations

The study was reviewed under the Expedited designation and approved by the institutional review board at Johns Hopkins University under protocol IRB00276421. Because this study was of minimal risk, participants consented with a waiver of documentation of consent that covered key study information, study purpose, procedures, risk and benefits, voluntary participation, payment, and study personnel contact information. Study data were anonymous. Amazon MTurk worker participants were paid US $3 for completing the survey. Qualtrics Panels’ participants were compensated based on prior compensation preferences (for example, participants can indicate that they would like to be compensated for SkyMiles, retail points, cash, or gift cards). Qualtrics Panels participants were informed of their compensation when invited to take the survey. Data collection was conducted from June 2022 through March 2023.

We completed a STARE-HI (Statement on Reporting of Evaluation Studies in Health Informatics) checklist and included the completed checklist in Multimedia Appendix 4 [34,35].

### Data Collection Procedures

We were interested in studying the user experience (UX) of the 2 FHx tools. The importance of UX (including usability) in technology acceptance is well recognized according to existing theoretical models (eg, Technology Acceptance Model and Task-Technology Fit) [36,37]. The assessment survey used to study UX described in Multimedia Appendix 3 collected quantitative data used to study the usability of the FHx data collection tools, the usefulness of the reports downloaded from those tools, and preferences to prioritize proposed chatbot features. It also collected qualitative data via free-text comments in response to questions about FHx tool features they liked or disliked. To study engagement, we passively collected log data exported from Juji and Qualtrics (for KIT and the form-based FHx data collection intervention, respectively).

First, to assess the usability of KIT and the form-based method, we analyzed assessment survey items based on the standardized System Usability Scale (SUS) [36] and the Chatbot Usability Questionnaire (CUQ) [38]. The SUS is a 10-question survey with a 5-point Likert response scale, ranging from “strongly disagree” to “strongly agree.” We adapted the SUS for our use case by removing 1 question (question 1: “I think I would like to use this system frequently”) that was not relevant to our context; the SUS score calculation was modified accordingly [39]. To capture chatbot users’ feedback, we included 16 items from CUQ in the assessment survey only for those participants randomized to the KIT intervention.

Second, we used 1 assessment survey item to assess the usefulness of reports downloaded from the FHx data collection tools for primary care visits. That item was “Do you think the final report from KIT would be useful to share with your primary care provider?” This was measured on a 5-point Likert scale.

Third, we aimed to assess participant interactions with the FHx data collection tools to understand if there were differences in UX and duration of experience between the tools (ie, engagement measures). We used log data on the duration of tool use, resource use (ie, button clicks and questions asked), and several conditions reported to measure engagement quantitatively.

Fourth, to supplement the quantitative data collected to study tool usability, report usefulness, and engagement, we included 2 free-text questions in the assessment survey about which features they liked or disliked in the collection tool they used. The free text was intended to capture a more comprehensive understanding of usability, report usefulness, and engagement that could not be captured through quantitative data alone. For example, for engagement, we could collect data on feelings associated with the FHx tool (such as interest and involvement) that went beyond the log data in the engagement quantitative data.

Finally, to assess priorities to enhance KIT in future iterations, we asked users to rate 3 chatbot-specific features that, if included in future chatbot design, would improve FHx collection. These features could potentially improve chatbot UX and value to users in health care contexts, as demonstrated by previous studies [40-45]. The included chatbot-specific features (with potential examples) were chatbot personalization (providing more education or tailored responses based on user entries), media elements (photos, videos, and graphics interchange formats as chatbot responses), and gamification (point reward system for engaging with the chatbot, complete FHx collection, and sharing results with family). For each feature, the participants picked 1 of 3 response options (high, medium, or low priority).

### Mixed Methods Data Analyses

For the quantitative analyses, we compared differences in mean *usability*, *engagement*, and *usefulness* measures between the 2 FHx data collection tools using data from all study participants who passed data quality checks. Participants were removed from the final analysis if they had low-quality UX comments data. The significance of the differences was assessed with the Welch 2-sample 2-tailed *t* test with a 95% CI. For individuals randomized to KIT, we reported the number and percentage of individuals who agreed (“strongly agree” or “agree”), disagreed (“strongly disagree” or “disagree”), and responded neutrally to each item from the CUQ. As an exploratory data analysis, we performed multiple linear regression to explore whether participants’ engagement with the FHx tools (as measured from log data) influenced their reported SUS usability scores. For 3 proposed chatbot feature enhancements (gamification, media elements, and personalization), we assessed *enhancement prioritization* based on the frequency of individuals indicating a high or medium priority from the largest (rank 1) to the smallest.

For the qualitative data analyses of *usability*, *engagement,* and *usefulness*, the 3 research team members (MHN, JS, and COT) used a set of definitions for constructs relevant to deductive coding. The definitions were aligned with the quantitative metrics captured for this study. They were further adapted for qualitative analysis by drawing from factors in measurement tools in human-computer interaction and information technology design settings [38,46,47]. For example, the definition used for usability-related comments captured “effectiveness (ability of users to complete tasks using the system, and the quality of the output of the tasks), efficiency (level of resource consumed in performing tasks), and satisfaction (users’ subjective reactions to using the system),” derived from the SUS definition of usability [38]. In addition, 2 coders mapped free-text comments shared by study participants on usability, engagement, and usefulness constructs. When concordance between the 2 coders was not achieved, the third research team member served as arbiter. We also noted comments on *desired features* made by both intervention groups (KIT and form). After we reached a consensus on theme categorization, author MHN reviewed comments within each theme to organize the data for subthemes and key concepts. Counts were reported for comments categorized under each subtheme and concept.

All quantitative analyses were conducted using R (version 4.2.0; R Foundation for Statistical Computing), and qualitative data were analyzed in Microsoft Excel (Microsoft Corp). In the last stage of the analysis, the qualitative responses relevant to usability, engagement, and report usefulness were compared with findings from the quantitative analyses. Doing this provided additional context for our quantitative findings.

## Results

### Study Population

A total of 138 MTurk workers and 445 Qualtrics Panel participants were randomized to an FHx data collection intervention.

Of these, 38 (27.5%) MTurk workers and 332 (74.6%) Qualtrics Panel participant responses were not included because they had duplicate responses, knowledge check failures, or failed to complete the study. Details on the Qualtrics Panels sample exclusions can be found in the in Multimedia Appendix 5. Table 1 summarizes self-reported demographic information. There were no significant differences between the form and KIT populations. The total cohort was primarily composed of female participants (109/213, 51.2%), aged ≥45 years (127/213, 59.6%), non-Hispanic or Latino White (164/213, 77%), and with a bachelor degree or higher (108/213, 50.4%). The largest share of participants was located in the Southeast region of the United States (69/213, 32.4%).

**Table 1:** Participant characteristics of the total cohort—Amazon Mechanical Turk and Qualtrics panels.

			Total (N=213), n (%)		Form based (n=112), n (%)		KIT^a^ (n=101), n (%)		*P* value	
**Gender**										.30
	Women	109 (51.2)		52 (46.4)		57 (56.4)				
	Men	100 (46.9)		57 (50.9)		43 (42.6)				
	Nonbinary	2 (0.9)		1 (0.9)		1 (1)				
	Prefer not to say	2 (0.9)		2 (1.8)		0 (0)				
**Age (y)**										.23
	≤44	84 (39.4)		39 (34.8)		45 (44.6)				
	≥45	127 (59.6)		71 (63.4)		56 (55.4)				
**Race**										.44
	Non-Hispanic or Latino White	164 (77)		86 (76.8)		78 (77.2)				
	Other ethnicities or race	44 (20.7)		22 (19.6)		22 (21.8)				
	Missing	5 (2.3)		4 (3.6)		1 (1)				
**Region**										.51
	Midwest (Iowa, Illinois, Indiana, Kansas, Michigan, Montana, Missouri, North Dakota, Nebraska, Ohio, South Dakota, and Wisconsin)	41 (19.2)		25 (22.3)		16 (15.8)				
	Northeast (Connecticut, District of Columbia, Delaware, Massachusetts, Maryland, Maine, New Hampshire, New Jersey, New York, Pennsylvania, Rhode Island, and Vermont)	36 (16.9)		19 (17)		17 (16.8)				
	Southeast (Alabama, Arkansas, Florida, Georgia, Kentucky, Louisiana, Mississippi, North Carolina, South Carolina, Tennessee, Virginia, and West Virginia)	69 (32.4)		32 (28.6)		37 (36.6)				
	Southwest (Arizona, New Mexico, Oklahoma, and Texas)	26 (12.2)		16 (14.3)		10 (9.9)				
	West (Alaska, California, Colorado, Hawaii, Idaho, Montana, Nevada, Oregon, Utah, Washington, and Wyoming)	41 (19.2)		20 (17.9)		21 (20.8)				
**Education**										.64
	Bachelor’s or higher	108 (50.7)		57 (50.9)		51 (50.5)				
	Lower than a bachelor’s degree	101 (47.4)		53 (47.3)		48 (47.5)				
	Prefer not to say	1 (0.5)		1 (0.9)		0 (0)				

^a^KIT: the curious interactive test.

Table 2 provides a quantitative analysis of summary statistics of mean and median usability, engagement, and usefulness measures according to the FHx data collection intervention type. All following results tables exclude participants from analysis who had low-quality UX comments results –4/101 KIT participants and 4/112 form-based participants.

**Table 2:** Combined cohort results for usability, engagement, and usefulness outcomes for KIT^a^ as compared with the form-based family health history tool.

Outcome and measure				Intervention type					*P* value
				Form based (n=108)		KIT (n=97)			
**Usability**									
	**SUS^b^ scores**								<.001^c^
		Mean (SD)	61.9 (9.16)		80.2 (17.6)				
		Median (IQR)	61.1 (58.3-72.2)		86.1 (66.6-94.4)				
	**CUQ^d^ scores**								N/A^f^
		Mean (SD)	—^e^		79.4 (13.6)				
		Median (IQR)	—		81.3 (1.50-4.70)				
**Engagement**									
	**Interaction duration**								.04^g^
		Mean (SD)	7.97 (8.30)		5.90 (5.79)				
		Median (IQR)	5.90 (3.85-8.38)		4.00 (3.00-7.00)				
	**Responses per minute**								.85
		Mean (SD)	3.26 (2.26)		3.21 (1.91)				
		Median (IQR)	2.93 (1.29-4.66)		3.14 (1.50-4.70)				
	**Number of reported conditions**								.04^g^
		Mean (SD)	10.1 (7.53)		7.77 (8.55)				
		Median (IQR)	9.00 (5.00-14.00)		6.00 (2.00-9.00)				
	**Character length of comments about liked features**								.40
		Mean (SD)	62.7 (41.3)		67.5 (40.6)				
		Median (IQR)	55.5 (35.0-82.0)		59.5 (39.0-86.5)				
	**Character length of comments about disliked features**								.88
		Mean (SD)	65.4 (50.9)		66.4 (42.1)				
		Median (IQR)	51.5 (33.0-83.0)		53.0 (37.0-84.0)				
**Usefulness**									
	**Perceived usefulness of final report**								.13
		Mean (SD)	4.29 (0.865)		4.08 (1.05)				
		Median (IQR)	4.00 (4.00-5.00)		4.00 (4.00-5.00)				

^a^KIT: the curious interactive test.

^b^SUS: System Usability Score.

^c^*P*<.001.

^d^CUQ: Chatbot Usability Questionnaire.

^e^Not applicable for the form-based group.

^f^N/A: not applicable because the form-based group did not complete the Chatbot Usability Questionnaire.

^g^*P*<.05.

Tables 3 and 4 summarize the qualitative analysis of positive and negative comments mapped to usability, engagement, and usefulness concepts for each FHx data collection intervention. Table 3 summarizes form-based qualitative results, and Table 4 summarizes KIT qualitative results.

**Table 3:** Form: themes and subthemes from qualitative analysis of form-based user experience comments^a^.

Interventions, themes, and subthemes, and concepts (+/–; n)				(+/–) Example comments
**Usability**				
	**Ease of use (53)**			
		Ease of use (+; 44) Ease of understanding (+; 7) Difficult to navigate (–; 1) Too technical (–; 1)	+: “It was easy and fast. I felt like I was able to quickly and accurately input the info as someone who already was confident in their family medical history.” –: “The method to select the health issue could be more explicit; use a check box.”	
	**Specific functionality (5)**			
		Unclear or inconvenient functionality (–; 5)	–: “On each page I could still select ’None of the above’ even if I selected a disease”	
**Usefulness**				
	**Report-related comments (8)**			
		Can be shared with family or family would use (+; 2) Report allows you to see family history in total (+; 2) Report is not useful (–; 1) Report is too long (–; 1) Report may not be accurate (–; 1) Report download does not work (–; 1)	+: “I liked that the tool was simple to use. It was also easy to read, and I could see my other family members using it as well.” +: “The report should summarize the results in a nice concise format, as opposed to the lengthy questionnaire.” –: “Tried to download collection tool and it didn’t work.”	
**Engagement**				
	**Overall design and organization (41)**			
		Comprehensive (+; 4) Conditions are provided (+; 3) General positive comments about interface (+; 10) Awkward user interface (–; 1) Condition list is not comprehensive enough (–; 10) Intrusive (–; 2) Too long (–; 3) Quick (+; 8)	+: “They cover a very comprehensive range of content that is easy to use” +: “The answers were already there. I just had to choose from the available options instead of thinking of each possible disease myself. It was straightforward. Very easy and unobtrusive.” +: “I like the feature that each disease is asked separately instead of being cluttered in a table.” –: “I kept having to go back and forth to make my answers correct because I kept realizing how I was clicking it incorrectly. I’m highly computer savvy and this was awkward.”	
	**Comments about specific functionality (43)**			
		Information buttons (+; 13) Multiple-choice format (+; 6) Desire for additional information (–; 5) Desire for additional functionality (ie, to add more detailed information; –; 7) No self-reporting included (–; 12)	+: “I liked that it was easy and there wasn’t any ambiguity. I also liked the info buttons so you could read about the disease if you weren’t sure what it was.” –: “The person completing the survey tool could not insert information about themselves.” +: “No typing required; mouse click only was easy.” –: “I didn’t like that there were no ways to answer further.” –: “I would have liked to report more information when reporting the diseases my grandparents had. I felt like I needed to put in more information or would have liked to explained more, such as some of the conditions were the result of having surgery.”	

^a^Count is not mutually exclusive (individual likes and dislikes can be related to multiple themes).

**Table 4:** KIT^a^: Themes and subthemes from qualitative analysis of KIT user experience comments.

Interventions, themes, subthemes, and concepts (+ or –; n)				(+ or –) Example comments
**Usability**				
	**Ease of use (44)**			
		General ease of use (+; 30) Ease of understanding (+; 4) Quick (+; 10)	+: “I liked that it listed out the different conditions to click, rather than having me type them in since medical terminology can be difficult to spell. Overall, I just liked how easy it was to use.” +: “I liked the speed of it. I liked how easy it was to answer her questions and some of the suggestions to her questions were nice also.”	
**Usefulness**				
	**Report-related comments (10)**			
		Transcript is difficult to read (–; 7) Download functionality issues (–; 1) Unclear purpose (–; 1) Desire for personalized response (–; 1)	–: “The final report was a bunch of gibberish and unreadable.” –: “I was unsure how exactly this information would be used relative to my family history.” –: “I thought the questions were somewhat generic, and no information based on my responses was provided.”	
**Engagement**				
	**Chatbot personality (27)**			
		Conversational (+; 1) Engaging (+; 1) Nonpersonal (+; 1) Understanding (+; 1) Able to elicit honest responses (+; 1) Informative (+; 6) General positive comments about personality (+; 6) Robotic personality (–; 10)	+: “...I like that it was generally ‘friendly’ acting, but still had the distance of being a chat bot, so I felt less awkward about my answers/potential for mistakes, etc” +: “Engaging, thorough clarification by Kit and very easy to ask questions” –: “The bot seem very impersonal and formal.”	
	**Overall design and organization (56)**			
		Conditions are provided (+; 17) Multiple-choice buttons (+; 8) FHxc collection is guided (+; 5) Not comprehensive enough (–; 1) Functionality issues (–; 3) Desire for additional information (–; 10) Lack of functionality to add more details (–; 7) Desire for personalized response (–; 5)	+: “I liked to see all the options for the health problems. I was able to recall family problems that I initially didn’t think of.” +: “I felt that the multiple-choice questions were good and provided a safe space to be truthful about personal family business.” +: “I felt it guided me through collecting a family history quite well; it was easy to focus on one relative and disease at a time.” –: “KIT kept freezing and I had to refresh 3 or 4 times to reactivate her.” –: “I think descriptions of what each disease was on hover would be useful, or more options to choose from.” –: “I wish there would have been a little more info about the health conditions and my risks provided while we talked.”	
	**Conversation pace (19)**			
		Quick (+; 10) Too fast (–; 6) Too slow (–; 3)	+: “Able to answer my questions quickly” – “It felt like it moved fast, and even though I could have scrolled up/down, delayed answering, etc. I still felt like I was being rushed through the process.” – “There was a lot of information to read at once that was sent quickly and I had to scroll up to start reading at the beginning.” – “It seemed a little slow to respond.”	

^a^KIT: the curious interactive test.

### Usability

The quantitative usability analyses showed a significant difference (*P*<.001) in median SUS scores, with a median score of 61.1 for form respondents and 86.1 for KIT participants (Table 2). Both the quantitative CUQ questionnaire results and the qualitative findings gave insights into the positive features contributing to SUS scores.

Aligning with KIT participants’ high SUS scores, KIT users reported a mean CUQ score of 79.4 (SD 13.6). Findings from analyzing CUQ questionnaires (Table 5) indicated the high (positive) scores for questions related to the ease of use (90/97, 93% agreed with P8 and 81/97, 84% disagreed with N8), ease of navigation (90/97, 93% agreed with P4 and 81/97, 84% disagreed with N4), ability to answer (75/97, 77% agreed with P6 and 81/97, 84% disagreed with N6), and understanding user prompts (78/97, 80% agreed with P5 and 82/97, 85% disagreed with N5). Similarly, the qualitative analyses (Form: Table 3 and KIT: Table 4) indicated that both KIT and form users valued the ease of use, citing ease of understanding and speed as positive attributes of the 2 methods. For example, one respondent noted:

I liked the speed of it. I liked how easy it was to answer her questions, and some of the suggestions to her questions were nice also.

Table 4

Qualitative findings clarified negative issues affecting usability, showing that form users noted the form being unclear or having inconvenient functionality (5 respondents; eg, “The method to select the health issue could be more explicit; use a check box”), being difficult to navigate (1 respondent), and being too technical (1 respondent; Table 3). For the KIT method, no negative features were noted as comments for usability.

**Table 5:** CUQ^a^ responses by item^b^ (n=97).

CUQ item number		CUQ item	Agree, n (%)	Neutral, n (%)	Disagree, n (%)
**Positive constructs**					
	P1	KIT’s^c^ personality was realistic and engaging	56 (58)	32 (33)	9 (9)
	P2	KIT was welcoming during the initial setup	77 (79)	16 (17)	4 (4)
	P3	KIT explained its scope and purpose well	51 (53)	14 (14)	32 (33)
	P4	KIT was easy to navigate	90 (93)	6 (6)	1 (1)
	P5	KIT understood me well	78 (80)	16 (17)	3 (3)
	P6	KIT’s responses were useful, appropriate, and informative	75 (77)	17 (18)	5 (5)
	P7	KIT coped well with any errors or mistakes	44 (45)	42 (43)	11 (11)
	P8	KIT was very easy to use	90 (93)	3 (3)	4 (4)
**Negative constructs**					
	N1	KIT seemed too robotic	60 (62)	20 (21)	17 (18)
	N2	KIT seemed very unfriendly	46 (47)	7 (7)	44 (45)
	N3	KIT gave no indication as to its purpose	92 (95)	3 (3)	2 (2)
	N4	It would be easy to get confused when using KIT	81 (83)	7 (7)	9 (9)
	N5	KIT failed to recognize a lot of my inputs	82 (85)	8 (8)	7 (7)
	N6	KIT’s responses were not relevant	81 (84)	13 (13)	3 (3)
	N7	KIT seemed unable to handle any errors	63 (65)	27 (28)	7 (7)
	N8	KIT was very complex	81 (84)	9 (9)	7 (7)

^a^CUQ: Chatbot Usability Questionnaire.

^b^Items were measured on a 5-point Likert scale. Agree and disagree responses are grouped.

^c^KIT: the curious interactive test.

### Engagement

Findings from analyzing engagement were mixed. A subset of the CUQ items used to assess KIT captured engagement-related concepts (Table 5).

In particular, we found that users responded more neutrally or negatively to CUQ questions related to KIT’s onboarding (14/97, 14% were neutral and 32/97, 33% disagreed with P3; less neutral or negative sentiment with N3), error management (42/97, 43% were neutral and 11/97, 11% disagreed with P7; 27/97, 28% were neutral and 7/97, 7% agreed with N7), and KIT’s personality (33/97, 33% were neutral and 9/97, 9% disagreed with P1; 20/97, 21% were neutral and 17/97, 18% agreed with N1; 7/97, 7% were neutral and 44/97, 45% agreed with N2), as presented in Table 5. User comments about error handling and personality were also present in the qualitative data. Users mentioned functionality issues (3 respondents; Table 4), such as KIT crashing, which might add context to the CUQ error handling questions (CUQ items P7 and N7).

In addition, participants shared a mix of positive and negative sentiments about KIT’s personality. In total, 10 KIT respondents identified KIT’s robotic personality in the qualitative findings, for example, remarking that “The bot seemed very impersonal and formal,” which aligns with the neutral or negative CUQ finding for personality questions (CUQ items P1 and N1). However, most (17/27, 63%) user comments related to KIT’s personality were positive, contrasting with the CUQ finding. One such comment mentioned, “...I like that it was generally ‘friendly’ acting, but still had the distance of being a chat bot, so I felt less awkward about my answers/potential for mistakes, etc.,” in which they found the nonpersonal nature of KIT to be a positive feature. In total, 2 form users felt that the questions were intrusive, which was not a comment found in KIT users’ responses.

We quantitatively compared engagement log data between KIT and the form-based method and found significant differences in 2 measures, interaction duration (mean of form 7.97 minutes, KIT 5.90 minutes; *P*=.04) and the number of reported conditions (mean of form 10.1, mean of KIT 7.77; *P*=.04), as presented in Table 2. We examined user comments for features that influenced interaction time or speed, contributing to the significant quantitative findings. From the qualitative data, we found that 6 (6%) of the 97 KIT participants felt the conversational pace of KIT was too fast with 1 respondent noting, “It felt like it moved fast, and even though I could have scrolled up/down, delayed answering, etc. I still felt like I was being rushed through the process” (Table 4). The conversational pace of KIT, as identified from the qualitative data, may have contributed to the significant quantitative finding in interaction duration.

Both KIT and form users said they wanted additional features or functionality within the FHx data collection tools. In total, 5 (4.6%) of the 108 form participants and 10 (10%) of the 97 KIT participants wanted the FHx tool to present additional information (example from a KIT participant, “I think descriptions of what each disease was on hover would be useful, or more options to choose from”). In addition, 7 (7%) form participants and 7 (7%) KIT participants expressed the desire for additional functionality. For form users, examples of this functionality included adding other family members to the history record (ie, aunts and uncles, “...I think we should add Father and Mother’s Siblings”) and adding more details surrounding a family member or condition, that is, specify which side of the family for a grandparent, specify how the condition occurred, or was diagnosed:

I would have liked to report more information when reporting the diseases my grandparents had. I felt like I needed to put in more information or would have liked to explained more, such as some of the conditions were the result of having surgery.

KIT users wanted the functionality to add more detailed and open-ended information about their conditions (eg, “I wish the choice of other allowed the consumer to type in that box”) and to add more details about their family members, like form users (eg, “there could have been more specifics on which family member”).

Some desired features were unique to form or KIT participants. In total, 12 (11%) of the 108 form participants expressed the desire to include themselves in the FHx record:

I thought it would have me listed too, which it didn’t. I was surprised I couldn’t put down what things I have or have had.

In total, 5 (5%) of the 97 KIT users expressed a desire for a personalized response from KIT, with 1 respondent mentioning, “I wish there would have been a little more info about the health conditions and my risks provided while we talked.”

As an exploratory analysis, to understand whether participants’ engagement with the FHx tools influenced their reported SUS usability scores, we performed multiple linear regression, including responses per minute, UX comments, likes and dislikes, length, duration, cohort (Qualtrics Panels and MTurk), and intervention type (KIT and form based) as covariates. Responses per minute were significantly correlated with SUS scores, β=1.193; t_198_=2.509; *P*=.02. Additional details are in Table S2 in Multimedia Appendix 5.

### Report Usefulness

Analysis of the usefulness of the final reports showed that both form and KIT respondents gave a median score of 4 in response to the Likert scale question regarding report usefulness, indicating that participants somewhat agreed that the final reports were useful. There was no significant difference found between groups, with mean scores of 4.29 for form-based users and 4.08 for KIT users (*P*=.13). The qualitative user comments from form users highlighted 2 features that may have made the form-based FHx report useful: the report could be shared with family members (2 respondents), and the report allowed you to see family history in total (2 respondents). There were no positive comments from KIT users in the qualitative data.

For both groups, the qualitative data provided more specific reasons why participants did not “strongly agree” (median score of 5) to the report usefulness question in the quantitative findings. Both user groups noted common issues with the functionality and general use of both FHx reports. For functionality, 1 participant from each FHx tool commented on the difficulty of downloading the reports. For general use, some participants desired FHx report features beyond a simple copy of their interaction. For example, 1 form user suggested a summary report format (eg, “The report should summarize the results in a nice concise format, as opposed to the lengthy questionnaire ”; Table 3). KIT respondents had more negative sentiments about the report’s readability (7 user comments), which did not appear in form participant responses. However, 1 form user commented that the report was too long. Unlike form users, KIT users also desired a personalized response and clearer purpose for the final report (2 comments in Table 4, eg, “somewhat generic and no information based on my responses was provided”), suggesting this would improve upon the current usefulness of the report. While KIT users had no positive comments about the report’s usefulness, some form users expressed that the report provided them with a snapshot of their family history and that they could imagine their family members using it as well (4 comments in Table 4).

### Enhancement Prioritization of Proposed Chatbot Features

**Figure 4:**
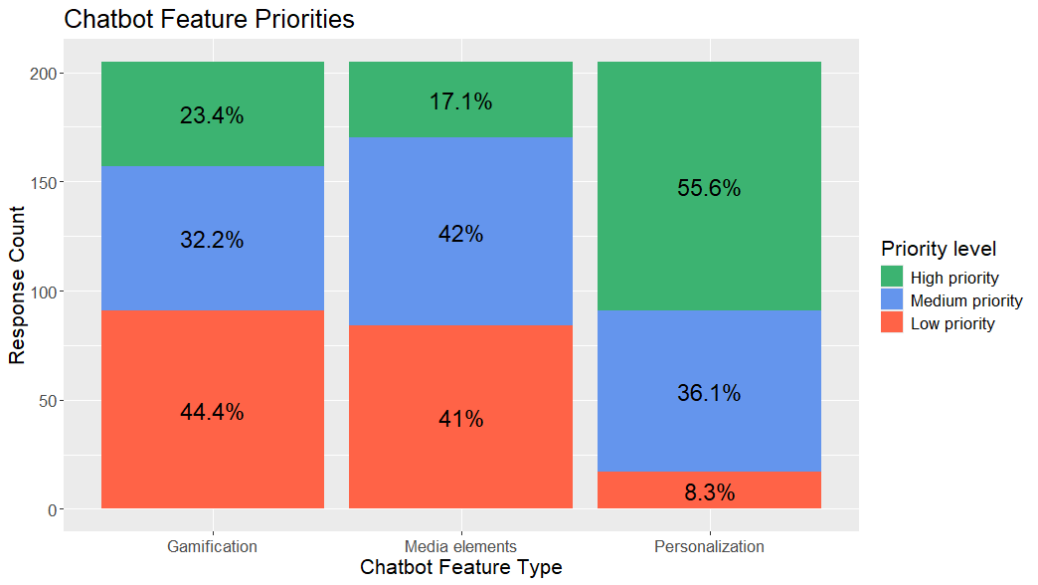
Prioritization of proposed chatbot feature enhancements: gamification, media elements, and personalization.

Both KIT and form-based users reported their opinions on proposed chatbot feature enhancements to prioritize for chatbot design. On the basis of the responses in Figure 4, they were ranked as follows: 1-personalization, 2-media elements, and 3-gamification. Notably, for all features, most respondents reported that they would prioritize them, with 55.6% (114/205), 60% (121/205), and 91.7% (188/205), indicating high or medium priority for gamification, media elements, and personalization, respectively.

## Discussion

### Principal Findings

Patient-facing digital health approaches to collect FHx are gaining acceptance and adoption due to their advantages over paper-based collection methods and their potential to support more convenient data collection, EHR integration, and automated risk assessment [13,48]. Currently, chatbots are being explored for clinical use as new ways to collect patient data and deliver care asynchronously, with use cases ranging from nutrition journaling to cognitive behavioral therapy delivery [43,44,49]. We purposefully designed our study to enable a fair comparison of KIT to the form-based FHx data collection. Both used the same data collection items and knowledge base for educational content (refer to the Intervention Design section). We focus our discussion on areas where our research provides insight into chatbots’ potential benefits over standard form-based methods and also discuss areas for enhancement. Where benefits exist, we further describe how specific features may improve chatbot usability and thus should be prioritized for future development.

Overall, we found that KIT was more usable than the form-based method (Table 2). There were some significant differences in 2 engagement metrics between the form-based method and KIT; the form elicited more reported conditions, and form users had longer interaction durations on average. We found no differences between KIT and the form-based method of FHx data collection for 3 engagement metrics (response per minute, character length of comments about liked features, and character length of comments about disliked features) and for the usefulness of the final report. Qualitative findings indicated that mismatches between what was implemented and expected functionalities may be an important factor distinguishing KIT from form-based FHx collection, with more respondents randomized to the form having more negative comments about this mismatch (eg, “On each page, I could still select ‘None of the above,’ even if I selected a disease”; Table 3). Further analyses of KIT indicated that participants felt it was easy to use and navigate, and they were able to understand user prompts and provide appropriate responses (Table 5). However, respondents were more neutral or showed a negative sentiment for KIT’s personality, onboarding process, and its management of errors. Despite this finding, qualitative findings indicated that when there were comments about KIT’s personality, they were positive, and in fact, its perceived nonhuman nature may be an added advantage (eg, “...I like that it was generally ‘friendly’ acting, but still had the distance of being a chat bot, so I felt less awkward about my answers/potential for mistakes, etc.”) We also proposed 3 areas to enhance KIT and found that all 3 (gamification, media elements, and personalization) were highly endorsed, with >50% (114/205, 121/205, 188/205, respectively) of the respondents indicating a high or moderate ranking for each of the features (Figure 4). According to our findings, the priority ranking of features was 1-personalization, 2-media elements, and 3-gamification.

Together, our findings provide valuable insight into design considerations for implementing a chatbot for FHx collection, including the potential benefit of its use over form-based methods. In the subsequent sections, we combine our findings with the broader literature exploring the use of conversational agents for FHx data collection.

### What Conversational Agents Can Add to FHx Data Collection

This study shows that conversational agents or chatbots offer specific functionality that may enhance FHx data collection and emphasize features to prioritize for future FHx tool development. We intentionally did not introduce many chatbot-specific features into KIT’s design to observe participants’ perceptions of a chatbot UX at a baseline level. Still, participants found KIT more usable than the form-based method (*P*<.001).

This distinction may be partly due to KIT’s conversational way of collecting FHx from users. Soni et al [26], for example, found that a chatbot used to complete a standardized health assessment was perceived as conversational, interactive, and intuitive, which may be important characteristics contributing to its usability. Most participants in that study reported a preference for a chatbot over a form-based strategy. In contrast to our work, however, they found no statistically significant differences in SUS scores when comparing the 2 groups [26]. In another study comparing a chatbot-based to a form-based survey format, Xiao et al [50] found that the chatbot elicited clearer, more relevant, and more specific responses than the form. In particular, the authors found higher levels of self-disclosure from users of the chatbot in comparison to form users, suggesting that chatbots may support higher-quality data collection by promoting interactive user engagement [50].

Another potential value of chatbots over other formats is their humanness. Despite limited chatbot-specific additions, a small portion of KIT users anthropomorphized KIT in their free-text comments, with about 18 participants referring to KIT as “she,” “her,” or by name in their comment (as opposed to “it,” or “the chatbot,” etc). This finding may reflect the KIT avatar we selected (feminine-presenting nurse). Participant self-disclosure may increase when chatbots have more humanization features applied to their design [51]. From a study of the 4th *All of Us* data set release, Sulieman et al [52] demonstrated that patient self-reported surveys contained data not found in the EHR and could help potentially fill in missing EHR data gaps [52]. Thus, we believe that designing FHx tools that encourage higher self-disclosure, such as chatbots, may further enhance the utility of patient-reported information to fill in gaps in EHR-documented information.

Comparing the form-based method with KIT, there are differences in the pathway to satisfy the users’ health information seeking. Chatbots allow for further questioning beyond a single info button. In a previous study comparing a FHx collection virtual conversational agent to the Surgeon General’s My Family History Portrait web tool, study participants preferred the dialogue or chat-based guidance of the chatbot over the standard web tool [53]. Chat-based guidance may be better equipped to support additional just-in-time health information seeking than an info button would. Health information seeking has been linked to more frequent discussions of FHx between patients and their families and with their primary care providers [54]. Better support for patients’ health information seeking needs within the FHx collection tool could encourage patients to be more likely to discuss FHx inside and outside the clinic.

Both form and KIT users responded positively to the multiple-choice button format, in which users were presented with answer options and could simply click the relevant answer options for data entry. This suggests that despite the chatbot format, users may still appreciate being shown response options, and this could reduce some working memory requirements for respondents and may be useful for medical history-taking for more accurate patient recall, drawing principles from cognitive load theory [55]. In a previous study of chatbot input mechanism (buttons vs free-text responses) on perceived pragmatic and hedonic interaction quality, chatbots with buttons were associated with both high usability (pragmatic quality) and engagement (hedonic quality) in comparison to chatbots accepting free-response answers only [56]. Moreover, button-input chatbots were not associated with less perceived anthropomorphism or social presence of the chatbot [56].

For both formats, users wanted the ability to answer further depending on the options they selected and to record medical history for themselves as well. These limitations stemmed from implementing the *All of Us* Family History Questionnaire outside of the *All of Us* Program context, as this family history questionnaire was designed to be administered in addition to other basic history surveys and EHR data integration [57]. However, for a stand-alone FHx tool, supporting users’ additional information would be beneficial. Chatbots may be especially capable of supporting further open-ended questions based on user input [50].

Both KIT and form users appreciated the information provided in the FHx tools. This adaptation to the *All of Us* questionnaire was not present in the original. By providing users with educational information about unfamiliar diseases, we sought to support their health literacy, potentially leading to more accurate data collection. Although there is limited literature on the effect of low health literacy on the accuracy of patient-reported health data, there is a great deal of evidence that low health literacy is linked to poorer health outcomes [58-60].

### The Final Report Is an Opportunity to Add Value to Patients

User feedback about the value of the FHx report was mixed. Although quantitative findings were positive, from qualitative user comments, we have found that the usefulness of the reports was limited by their awkward formatting, unclear purpose, and lack of value added beyond the scope of the data collection intervention (eg, summary table and personalized response). From this, we discuss motivations and design considerations for developing future FHx reports to add value for patients.

We were motivated to explore FHx report usefulness because of information gaps for both patients and providers. We sought to understand the value of FHx and to support the actionable use of FHx in care and condition management. In addition, we sought to better understand user desires for reports based on evidence that other FHx tools have developed patient-facing or clinician-facing reports. From a review of 62 generic and cancer-specific FHx tools, Cleophat et al [11] found that about one-fourth of FHx tools provided patient-facing recommendations after data collection, and about one-tenth of tools provided clinician-facing management recommendations. While some available FHx tools only provide clinician-facing decision support or no guidance at all, many provide patient-facing decision support through recommendations or risk assessments based on collected FHx data [11,12]. However, few studies highlight what specific features of FHx reports are valuable to patients.

Our work adds to the literature to identify FHx report features to improve usefulness for a patient’s primary care visit. For form users, their final report was a summary of their responses that they could choose to download. For KIT users, their final report was a time-stamped transcript of their responses as a text file they could download. We relied on the host interface’s (Qualtrics and Juji) baseline capabilities for both web-based tools without custom formatting.

In future FHx tool design, it is important to introduce what the use of the final report might be at the beginning of the intervention, as well as to customize it for consumer use intentionally and to understand patient preferences and attitudes toward features, such as personalized FHx risk assessment. Previous work has designed patient-facing genetic test reports to maximize patient understanding [61,62], and many of the lessons learned in the structure, content, communication style, and visual design of these reports can be applied to patient-facing FHx reports.

### Balance Between Participant Burden and Data Quality With FHx Collection

Variation in the onboarding process implemented in this study highlighted a balance between participant burden and the quality of FHx data collected. For the form, the only onboarding step was to review instructions. Participants were then presented with each of the 11 condition groups. Each condition group was asked to indicate whether any conditions within that group were present in their family, and if so, for what family members. They were asked this information regardless of whether they had any family members with a relevant history (Multimedia Appendix 1). For KIT, there were 2 onboarding steps. Following the instructions, users were presented with the initial list of 11 condition groups, from which they were prompted to select only those present (or known to be present) in their family. This process was modified based on KIT user comments during the pilot phase of this study (Multimedia Appendix 2) to eliminate the need to see irrelevant condition groups.

The number of reported conditions was significantly different between the 2 intervention groups, with form-based users reporting more conditions on average than KIT users, respectively (10.1 vs 7.77; *P*=.04). This finding suggests that differences in onboarding may have impacted the number of reported health conditions. While the onboarding strategy used in KIT may have reduced the burden for the users by decreasing the number of data items to collect, there was also a potential impact on the quality of FHx data collected. For example, there may have been instances where a specific condition name was known, but the condition group name was unknown. If the group name was not selected, the user would never see the specific condition found in their family. Therefore, the occurrence of missing FHx data could be higher. In future work, the chatbot onboarding strategy should be an important design consideration influencing data quality, and it could be particularly important when implementing chatbots for data collection.

### Opportunities for Large Language Models in FHx Collection

We learned that KIT’s flow-based nature may limit its interaction style. Large language models (LLMs) may help address some of the deficits identified in user comments. Personalization was the highest ranked among the 3 potential enhancements presented to participants. In addition, participants negatively responded to KIT’s personality, ability to explain its purpose, and ability to manage errors. The flow-based chatbot strategy we used did not customize error handling messages or its personality features; however, these issues may be addressed by integrating LLMs into future chatbot design. In a study exploring the use of LLMs to support chatbot collection of health self-reported data, Wei et al [63] present potential benefits of using an LLM-driven chatbot due to its abilities to ask specific follow-up questions, include social attributes, recover from errors, and track conversational context. For our work, we relied on a flow-based chatbot framework to compare the interfaces as directly as possible and observe any baseline chatbot characteristics that differentiate the usability of the 2 modalities. As LLMs and generative artificial intelligence continue to advance, they are being explored for use in health care contexts. However, there are still essential implementation considerations, such as Health Insurance Portability and Accountability Act compliance, model bias, and rigorous safety evaluation before widely deploying these tools for patient care [64].

### Additional Comparisons to Prior Work

This study assessed KIT’s usability among a crowdsourced cohort and not among in-clinic users. For health-specific contexts, prior work has not reached a consensus on whether chatbots are more usable or preferred compared to standard web-based forms in a clinical setting.

For clinician-facing use, Iftikhar et al [65] conducted a study to assess the comparative usability of 3 web-based forms (single-page form, multipage form, and chatbot [conversational form]) to record patient referrals to a cardiology department as replacement options for paper-based forms. In their study, the chatbot was the least preferred choice and had a below-average SUS score among participating nurses, commenting that the chatbot was difficult to understand and use. For patient-facing use, Te Pas et al [66] compared a chatbot with a standard web-based form to administer a preoperative questionnaire in an outpatient clinic setting [66]. They found that chatbot users reported higher User Experience Questionnaire scores than the standard form [66]. Chatbots are becoming more prevalent in modern life; therefore, the public may perceive chatbots to be more usable as they become more familiar. Although our crowdsourced findings suggest that KIT may be a more usable method than a standard form, further work is needed to understand how patients and care providers would perceive a FHx chatbot like KIT.

### Limitations

This study has some limitations. First, engagement metrics based on duration alone are not wholly informative of user involvement with a tool because they may measure inactive time. This limitation was partly addressed by including rate-based engagement measurements (responses per minute). Using responses per minute, we did not find any significant differences between form-based and KIT users.

Second, we introduced potential chatbot enhancements (personalization, media elements, and gamification) to all study participants. For form-based users, this question was more hypothetical than for KIT users, who responded to the question after chatbot interaction. Therefore, the question may measure different constructs between the groups. However, when conducting Welch *t* tests for response differences between groups, we observed no significant statistical differences (Multimedia Appendix 5).

Third, as described earlier, there were differences in the onboarding process between the KIT and form groups. These differences may have introduced some measurement bias, leading to the observed difference in the number of reported conditions between KIT and form users.

Fourth, we did not require participants to enter their true FHx, which limited a closer comparison of the quality of collected data between the 2 tools. Although an investigation into FHx data quality collected by web-based tools was not in the scope of this study, this is an important consideration for future FHx tool development and refinement.

Finally, some researchers have raised concerns about using crowdsourcing platforms for behavioral studies and health-related research, citing poor data quality, lack of research transparency, generalizability to other population groups, and potentially inflated outcomes [61-63]. In our study, we followed best practices for crowdsourcing study design and recruited from a vetted pool of MTurk participants. We also used Qualtrics Panels as a validation cohort. There may still, however, be a bias toward a sample with more familiarity with web-based data collection methods than a general population.

### Conclusions

We compared a flow-based chatbot (KIT) to a form-based method and observed that chatbot-specific features may contribute to a more usable experience. To support our comparison of the 2 methods, both incorporated FHx data collection items from the *All of Us* research program and the NLM MedlinePlus knowledge base for educational content. Our work contributes to the existing literature on how chatbots may be used for FHx collection and adds evidence of its potential benefit over form-based methods. In addition, findings from our study highlight opportunities to improve upon a final report summarizing the FHx collection experience, show that chatbot question prompts and question flow have an essential role in ultimately how complete the FHx data are, and show several areas that can be improved upon with a move to an LLM implementation strategy.

## Appendix

Multimedia Appendix 1 Intervention design.

Multimedia Appendix 2 Pilot results and modifications.

Multimedia Appendix 3 Assessment survey instrument.

Multimedia Appendix 4 STARE-HI (Statement on Reporting of Evaluation Studies in Health Informatics) checklist.

Multimedia Appendix 5 Additional results.

## Abbreviations

CUQ: Chatbot Usability Questionnaire
EHR: electronic health record
FHx: family health history
KIT: curious interactive test
LLM: large language model
MTurk: Mechanical Turk
NLM: National Library of Medicine
REDCap: Research Electronic Data Capture
STARE-HI: Statement on Reporting of Evaluation Studies in Health Informatics
SUS: System Usability Scale
UX: user experience

## Data Availability

The data sets generated during and analyzed during this study are not publicly available due to potentially identifiable information but are available from the corresponding author on reasonable request. The data generated and analyzed during this study are available from the corresponding author on reasonable request.
