# Supplementary material for: Usability, Engagement, and Report Usefulness of Chatbot-Based Family Health History Data Collection: Mixed Methods Analysis
Source: J Med Internet Res. 2024 Sep 30;26:e55164. doi: 10.2196/55164 (PMC11474129; doi:10.2196/55164)
Supplement: Multimedia Appendix 1 [file jmir_v26i1e55164_app1.docx]

## Multimedia Appendix 1 – Intervention Design


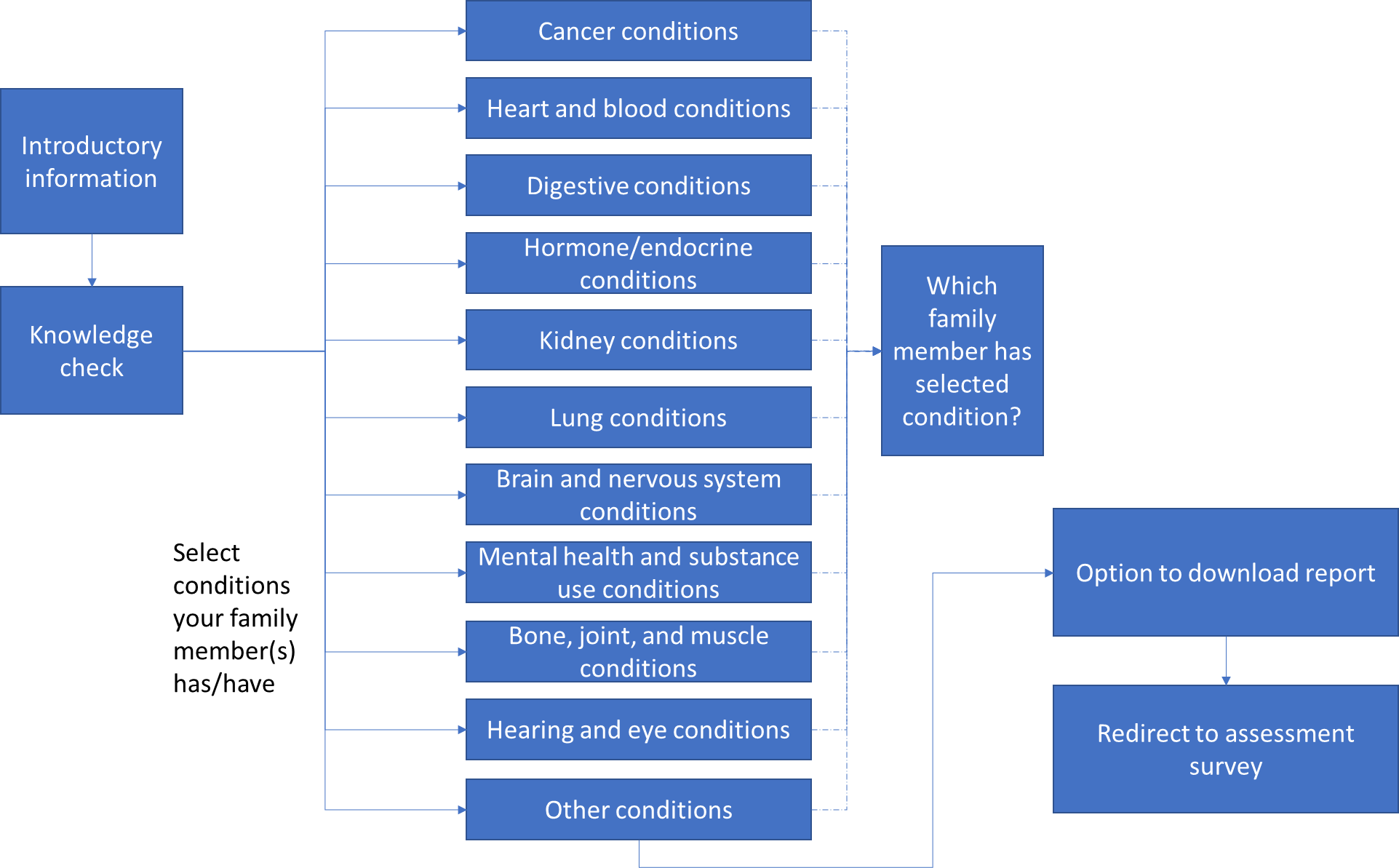


Figure S1A. Intervention question logic flow for form-based method

[Figure S2A. Form-based method questionnaire format and questions](https://livejohnshopkins-my.sharepoint.com/:b:/g/personal/mnguye79_jh_edu/ETkSf5EYQnVBku-K7_TB85UBx75zg7Ujo2WALpENn8ne0w?e=gJQcqz)


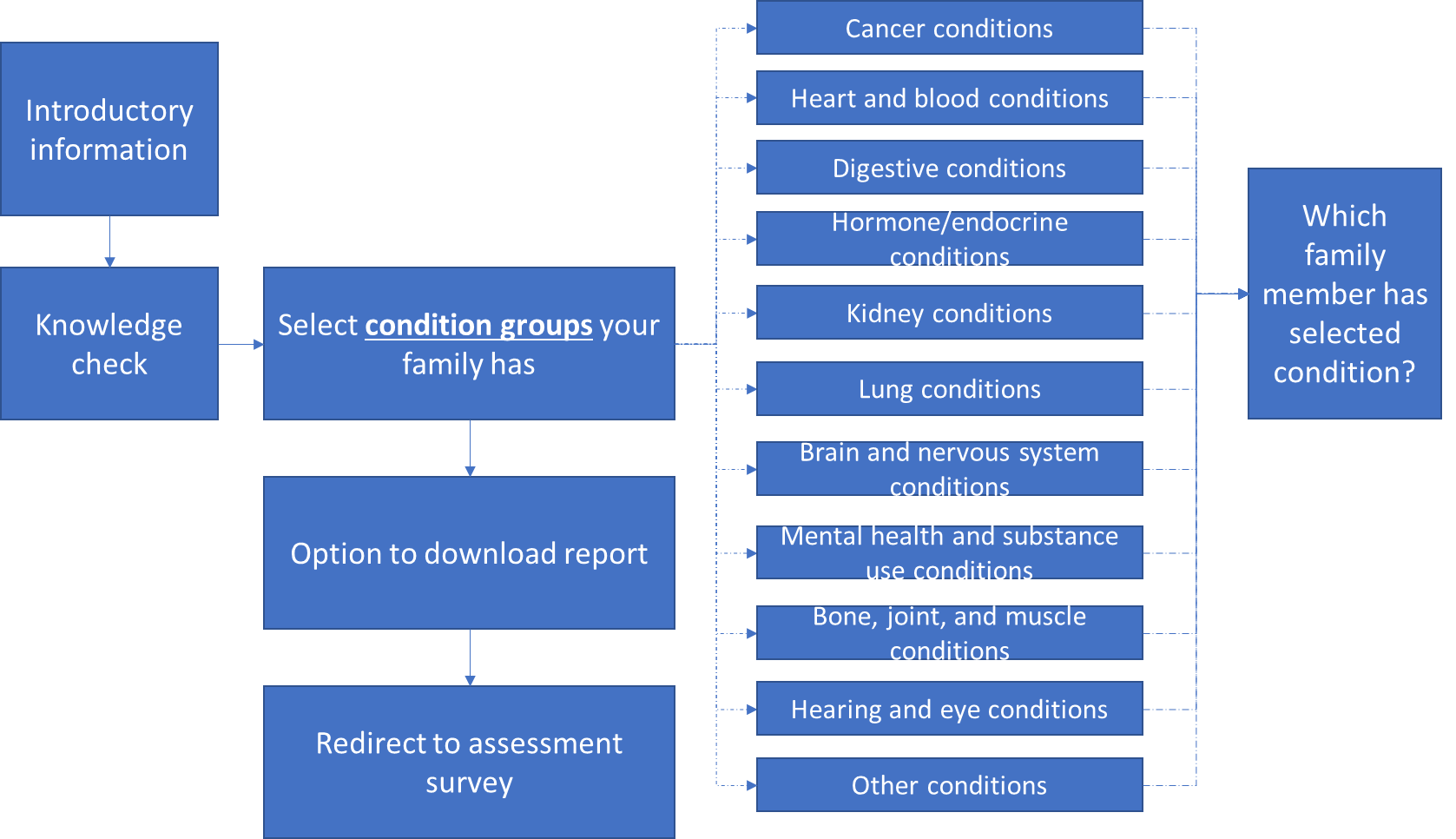


Figure 3A. Intervention question logic for KIT. Introduces new logic skip, “Select condition groups your family has,” not present in form

[Document S4A. KIT question format text file](https://livejohnshopkins-my.sharepoint.com/:t:/g/personal/mnguye79_jh_edu/EYITxLxDN3JKmYfK2PD1WUQBp8P2dHSyqv2fIhFsXIFENg?e=NGcyLy)

[Video S5A. KIT sample interaction](https://livejohnshopkins-my.sharepoint.com/:v:/g/personal/mnguye79_jh_edu/EVeeKMMhW2xKmY_lNzLUZZ4BLAAUKXzzbF3_pjWB3BKjJg?nav=eyJyZWZlcnJhbEluZm8iOnsicmVmZXJyYWxBcHAiOiJPbmVEcml2ZUZvckJ1c2luZXNzIiwicmVmZXJyYWxBcHBQbGF0Zm9ybSI6IldlYiIsInJlZmVycmFsTW9kZSI6InZpZXciLCJyZWZlcnJhbFZpZXciOiJNeUZpbGVzTGlua0NvcHkifX0&e=p1UBdo)
