# Supplementary material for: Usability, Engagement, and Report Usefulness of Chatbot-Based Family Health History Data Collection: Mixed Methods Analysis
Source: J Med Internet Res. 2024 Sep 30;26:e55164. doi: 10.2196/55164 (PMC11474129; doi:10.2196/55164)
Supplement: Multimedia Appendix 2 [file jmir_v26i1e55164_app2.docx]

## Multimedia Appendix 2 - Pilot results and modifications

In February 2022, we released a total of 6 batches (54 possible user entries) of the pilot HIT to Amazon MTurk. From this HIT release, we assessed 100 individuals for eligibility, of which 36 were eligible for the study. After intervention randomization, we yielded a final analysis cohort of 25 participants – 13 individuals for the form-based method, and 12 for KIT.

From the pilot study, there were several key lessons learned that were used to improve the MTurk and survey workflow as well as the FHx intervention, KIT. We added additional security measures to ensure participants were not accessing the consent form without first completing and fulfilling screening requirements. These changes included adding CAPTCHA tests before eligibility and consent forms and adding embedded data to Qualtrics redirect links to capture MTurk Worker IDs.

Interim Analysis – Amazon Mechanical Turk Cohort

For MTurk analysis, we performed data quality checks on duration and qualitative UX comment responses to ensure that users were properly completing the study and to assign bonuses where appropriate. We used interim SUS data to perform an effect size calculation with Cohen’s d.

Sample size was determined through interim data analysis. As a between-subjects study design, we used an independent t-test to compare differences in our outcome variables between the two FHx collection methods. To determine sample size, we performed a two-sample t-test power calculation in R using stats package function power.t.test with a choice of 80% power and a significance level of 0.05. We estimated the effect size by calculating Cohen’s d based on the interim SUS data we had collected after releasing 35 batches of MTurk HITs, each with 9 possible assignments each.

We reference best practices for SUS sample size calculation [42] and then calculated sample size based on the estimated variance of our SUS interim data with a desired confidence interval of 95% and a precision of +/-5 SUS points.

Interim Analysis – Qualtrics Panels Cohort

We conducted a soft launch with 20 respondents and then assessed interim data quality. As with MTurk interim analysis, we reviewed responses for data quality assurance based on respondent intervention duration and qualitative UX comments.

*Results of FHx tool design*

We implemented two changes to KIT based on user experience free response comments. Firstly, KIT’s latency in response time was reduced from the default setting of 5 seconds to 1 second in response to user comments noting that KIT took a long time to respond to their inputs. Secondly, pilot testers suggested that it might be beneficial to reduce the number of questions. Because of this, we added an initial skip logic question to KIT in which KIT asks users to select condition groups that they are aware their family members have first. Afterwards, users only answer questions for those relevant health condition groups. This logic adaptation was not made to the form-based method.
