# Supplementary material for: Usability, Engagement, and Report Usefulness of Chatbot-Based Family Health History Data Collection: Mixed Methods Analysis
Source: J Med Internet Res. 2024 Sep 30;26:e55164. doi: 10.2196/55164 (PMC11474129; doi:10.2196/55164)
Supplement: Multimedia Appendix 3 [file jmir_v26i1e55164_app3.pdf]

Intervention assessment survey –

Note: The form-based assessment survey and KIT assessment survey differed slightly in their contents. These differences are highlighted when appropriate throughout the document.

Usefulness Do you think the final report from KIT/FORM-BASED METHOD would be useful to share with your primary care provider?

- ○ Strongly disagree (1)
- ○ Somewhat disagree (2)
- ○ Neither agree nor disagree (3)
- ○ Somewhat agree (4)
- ○ Strongly agree (5)
- ○ N/A; I did not review the final report. (5)

Chatbot_features
We are exploring novel methods to improve family health history collection. A chatbot, which is a text-based conversational agent, is one method we are considering.
Please indicate your priority for the following functionality that chatbots can support.
OR
We are exploring ways to improve KIT.
Please indicate your priority for the following functionality that chatbots can support.

	High Priority (1)	Moderate Priority (2)	Low Priority (3)
Providing **more education or tailored responses** based on user entries (1)	○	○	○
**Media elements** (ie: photos, videos, gifs as chatbot responses) (5)	○	○	○
**Response gamification** (ie: point reward system for engaging with chatbot, completing family health history collection and sharing results with family) (2)	○	○	○

Highlighted text was only shown for KIT participants. Non-highlighted text includes more introduction of chatbots for form-based participants.

Usability For each of the following statements, please mark one box that best describes your reactions to the conversational agent KIT/FORM-BASED METHOD family health history collection tool.

	Strongly disagree (1)	Somewhat disagree (2)	Neither agree nor disagree (3)	Somewhat agree (4)	Strongly agree (5)
I found KIT/FORM-BASED METHOD **unnecessarily complex**. (2)	○	○	○	○	○
I thought KIT/FORM-BASED METHOD was **easy to use**. (3)	○	○	○	○	○
I think I would **need the support of a technical person** to be able to use KIT/FORM-BASED METHOD. (4)	○	○	○	○	○
I thought the various **functions** in KIT/FORM-BASED METHOD were **well integrated**. (5)	○	○	○	○	○
I thought there was **too much inconsistency** in KIT/FORM-BASED METHOD. (6)	○	○	○	○	○
I would imagine that most people would **learn** to use	○	○	○	○	○

KIT/FORM-BASED METHOD **very quickly**. (7)					
I found KIT/FORM-BASED METHOD **very cumbersome (awkward)** to use. (8)	○	○	○	○	○
I felt **very confident** using KIT/FORM-BASED METHOD. (9)	○	○	○	○	○
I **needed to learn a lot of things before I could get going** with KIT/FORM-BASED METHOD. (10)	○	○	○	○	○
KIT's personality was **realistic and engaging** (11)	○	○	○	○	○
KIT seemed **too robotic** (12)	○	○	○	○	○
KIT was **welcoming during initial setup** (13)	○	○	○	○	○
For this statement, select Somewhat disagree as your response. (28)	○	○	○	○	○

KIT seemed **very unfriendly** (14)					
KIT **explained its scope and purpose well** (15)					
KIT gave **no indication as to its purpose** (16)					
KIT was **easy to navigate** (17)					
It would be **easy to get confused** when using KIT (18)					
KIT **understood me well** (19)					
KIT **failed to recognize a lot of my inputs** (20)					
KIT's responses were **useful, appropriate, and informative** (21)					
KIT's responses were **not relevant** (22)					
KIT **coped well with any errors or mistakes** (23)					

KIT **seemed unable to handle any errors** (24)	○	○	○	○	○
KIT was very **easy to use** (25)	○	○	○	○	○
KIT was **very complex** (26)	○	○	○	○	○

Highlighted questions (CUQ) were only shown for KIT participants

like What features of the KIT/FORM-BASED METHOD family health history collection tool did you **like**? (20 characters minimum)

____

dislike What features of the KIT/FORM-BASED METHOD family health history collection tool did you **dislike**? (20 characters minimum)

____

background1 Please answer the following questions about your background:

gender What is your gender?

- ○ Male (1)
- ○ Female (2)
- ○ Other (3) ____
- ○ Prefer not to say (4)

---

english Do you speak English as your first language?

- ○ Yes (1)
- ○ No (2)
- ○ Prefer not to say (3)

---

ethnicity What is your ethnicity?

- ○ Hispanic or Latino (1)
- ○ Non-Hispanic or Latino (2)
- ○ Prefer not to say (3)

---

race What is your race?

- ☐ White or Caucasian (1)
- ☐ Black or African American (2)
- ☐ American Indian or Alaskan Native (3)
- ☐ Hawaiian or Pacific Islander (4)
- ☐ Asian or Asian American (5)
- ☐ Other (6) ____
- ☐ Prefer not to say (7)

---

age What is your age group?

- ○ 18-29 years old (1)
- ○ 30-44 years old (2)
- ○ 45-59 years old (3)
- ○ 60 years old or more (4)
- ○ Prefer not to say (5)

---

region What region of the United States do you live in?

- ◯ Midwest (IA, IL, IN, KS, MI, MN, MO, ND, NE, OH, SD, WI) (1)
- ◯ Northeast (CT, DC, DE, MA, MD, ME, NH, NJ, NY, PA, RI, VT) (2)
- ◯ Southeast (AL, AR, FL, GA, KY, LA, MS, NC, SC, TN, VA, WV) (3)
- ◯ Southwest (AZ, NM, OK, TX) (4)
- ◯ West (AK, CA, CO, HI, ID, MT, NV, OR, UT, WA, WY) (5)

---

education What is your highest level of education?

- ◯ Less than high school (1)
- ◯ High school graduate or GED (2)
- ◯ Some college (3)
- ◯ Bachelor's degree (4)
- ◯ Graduate or professional degree (5)
- ◯ Prefer not to say (6)

---

end Thank you for completing these questions. Clicking the submit button will direct you to your MTurk participant ID.

Note: This text was only present for MTurk participants. Qualtrics Panelists have generic submit text and thank you.

Page Break

End of Block: KIT/FORM-BASED METHOD
