# Supplementary material for: Usability, Engagement, and Report Usefulness of Chatbot-Based Family Health History Data Collection: Mixed Methods Analysis
Source: J Med Internet Res. 2024 Sep 30;26:e55164. doi: 10.2196/55164 (PMC11474129; doi:10.2196/55164)
Supplement: Multimedia Appendix 4 [file jmir_v26i1e55164_app4.pdf]

STARE-HI

Studies in Health Informatics: explanation and elaboration. Appl Clin Inform. 2013 Jul 24;4(3):331-58. doi: 10.4338/ACI-2013-04-RA-0024. PMID: 24155788; PMCID: PMC3799207.

the specific explanations for each section added as a column. We have also added a column to the checklist to add the section of our paper that covers the specified details where relevant.

Adapted from Table 1

Chapter in elaboration paper	STARE-HI item
**3.1**	**Title**
**3.2**	**Abstract**
**3.3**	**Keywords**
**3.4**	**Introduction**
3.4.1	Scientific background
3.4.2	Rationale for the study
3.4.3	Objectives of the study
**3.5**	**Study context**
Me	Organizational setting
3.5.2	System details and system in use
**3.6**	**Methods**
3.6.1	Study design
3.6.2	Theoretical background
3.6.3	Participants

3.6.4	Study flow
3.6.5	Outcome measures or evaluation criteria
3.6.6	Methods for data acquisition and measurement
3.6.7	Methods for data analysis
**3.7**	**Results**
3.7.1	Demographic and other study coverage data
3.7.2	Unexpected events during the study
3.7.3	Study findings and outcome data
3.7.4	Unexpected observations
**3.8**	**Discussion**
3.8.1	Answers to study questions
3.8.2	Strengths and weaknesses of the study
3.8.3	Results in relation to other studies
3.8.4	Meaning and generalizability of the study
3.8.5	Unanswered and new questions
**3.9**	**Conclusion**
**3.1**	**Authors' contribution**
**3.11**	**Competing interests**
**3.12**	**Acknowledgement**
**3.13**	**References**
**3.14**	**Appendices**

Description from elaboration paper	Item comprised in manuscript? (√/-)
"The title should give a clear indication of the type of evaluated system and the study question as well as the study design."	√
"The abstract must clearly describe the objective, setting, participants, measures, study design, major results, and conclusions."	√
"Among the keywords should be "evaluation" and keywords describing the type of system evaluated, the setting, outcome measures, and study design."	√
"Description of what is already known about the (type of) intervention that is the object of study, what are still open research questions, and why there is a need to answer them."	√
Short description of the motivation for the study; stakeholders and actors	√
The specific study questions and hypotheses, accompanied by permissions obtained in relation to the study.	√
The name, location and kind of health care facility and involved departments.	√
A description that enables the reader to understand how the system works (or is intended to work) and its phase in the system's life cycle.	√
The overall study design and the arguments for choosing it.	√
Theories – with appropriate references – on which the study is based and that guided the selection of the measurement instruments used.	√
Methods of selection of participating users, patients, units, hospitals, etc, including if applicable inclusion and exclusion criteria.	√

Details on date of beginning and end of the overall study and any study periods with clear descriptions of intervention.	√
Description of outcome measures used or other evaluation variables of interest together with definitions of key concepts.	√
Provide sufficient detail on data acquisition and measurement such that others are able to assess the appropriateness and any limitations, as well as to be able to replicate the measurement procedures of the study.	√
For quantitative data, state which statistical techniques were used for analysis. For qualitative data, indicate the analysis methods in detail. For all data analysis methods, indicate any software product used.	√
Baseline demographic data and clinical characteristics of study participants (users, patients, and units) and of the study.	√
Any unforeseen events that may have influenced the study results or outcome.	NA
Presenting the results of the study for each study question, for each outcome variable and evaluation criterion.	√
Any unintended (positive or negative) side-effects of the system that were not in focus at the study.	NA
A discussion of the answers identified versus the questions posed for the study.	√
Critical discussion of the methods used.	√
Make clear what exactly is novel about the obtained results.	√
Implication of the study findings, for the various stakeholders within the study and beyond.	√
Future research needs and opportunities.	√
Summary of the main findings, including the impact of the findings and how they relate back to the big picture provided.	√
Explicit description of the contributions of the authors to make sure that each author qualifies for authorship.	√
A statement of the interest, financial or otherwise, the authors may have with respect to the outcome of the study.	√
Acknowledgements of any financial or other support.	√
All references needed for the argumentation	√
Any supporting material, such as detailed descriptions of methods/tools (e.g. a questionnaire), specific data analysis techniques and detailed study results	√

**Section in manuscript**
Title
Abstract
After abstract
Introduction
Introduction
Introduction
Methods - Study design and population
Methods - Intervention design
Methods - Study design and population
Methods - Data Collection Procedures
Methods - Study design and population

Methods - Study design and population and Methods - Ethical Considerations
Methods - Data Collection Procedures
Methods - Data Collection Procedures
Methods - Mixed Methods Data Analyses
Results- Study Population
Results - Usability; Results- Engagement; Results- Report Usefulness; Results - Enhancement Prioritization of Chatbot Features
All discussion sections
All discussion sections
All discussion sections
All discussion sections
All discussion sections
Conclusion
Author's contributions
Acknowledgments
