# Supplementary material for: Usability, Engagement, and Report Usefulness of Chatbot-Based Family Health History Data Collection: Mixed Methods Analysis
Source: J Med Internet Res. 2024 Sep 30;26:e55164. doi: 10.2196/55164 (PMC11474129; doi:10.2196/55164)
Supplement: Multimedia Appendix 5 [file jmir_v26i1e55164_app5.docx]

## Multimedia Appendix 5 – Additional results


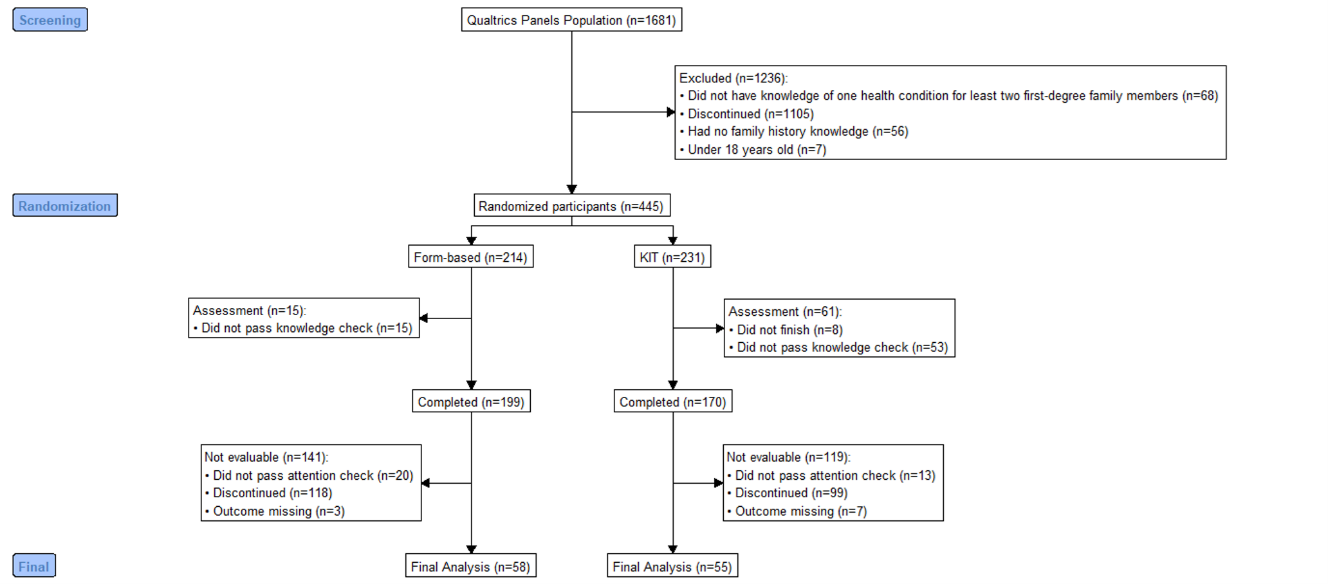


Figure S1D. Qualtrics Panels CONSORT Diagram

|  |  | Total | | Form | | KIT | | Form *P*-value | KIT  *P*-value |
| --- | --- | --- | --- | --- | --- | --- | --- | --- | --- |
|  |  | Mechanical Turk  (N = 100) | Qualtrics Panels  (N = 113) | Mechanical Turk  (N = 53) | Qualtrics Panels  (N = 59) | Mechanical Turk  (N = 47) | Qualtrics Panels  (N = 54) |  |  |
| Gender | Female | 50 (50.0%) | 59 (52.2%) | 25 (47.2%) | 27 (45.8%) | 25 (53.2%) | 32 (59.3%) | *P* = .203 | *P* = .305 |
|  | Male | 46 (46.0%) | 54 (47.8%) | 25 (47.2%) | 32 (54.2%) | 21 (44.7%) | 22 (40.7%) |  |  |
|  | Other | 2 (2.0%) | 0 (0%) | 1 (1.9%) | 0 (0%) | 1 (2.1%) | 0 (0%) |  |  |
|  | Prefer not to say | 2 (2.0%) | 0 (0%) | 2 (3.8%) | 0 (0%) | 0 (0%) | 0 (0%) |  |  |
| Age | 44 years old or younger | 52 (52.0%) | 32 (28.3%) | 28 (52.8%) | 11 (18.6%) | 24 (51.1%) | 21 (38.9%) | *P* <.001 | *P* <.001 |
|  | 45 years old or older | 46 (46.0%) | 81 (71.7%) | 23 (43.4%) | 48 (81.4%) | 23 (48.9%) | 33 (61.1%) |  |  |
| Race and Ethnicity | Non-Hispanic or Latino White | 73 (73.0%) | 91 (80.5%) | 38 (71.7%) | 48 (81.4%) | 35 (74.5%) | 43 (79.6%) | *P* = .416 | *P* = .363 |
|  | Other Ethnicity and/or Race | 24 (24.0%) | 20 (17.7%) | 12 (22.6%) | 10 (16.9%) | 12 (25.5%) | 10 (18.5%) |  |  |
|  | Missing | 3 (3.0%) | 2 (1.8%) | 3 (5.7%) | 1 (1.7%) | 0 (0%) | 1 (1.9%) |  |  |
| Geographic Region of the United States | Midwest | 18 (18.0%) | 23 (20.4%) | 13 (24.5%) | 12 (20.3%) | 5 (10.6%) | 11 (20.4%) | *P* = .333 | *P* = .14 |
|  | Northeast | 19 (19.0%) | 17 (15.0%) | 10 (18.9%) | 9 (15.3%) | 9 (19.1%) | 8 (14.8%) |  |  |
|  | Southeast | 37 (37.0%) | 32 (28.3%) | 19 (35.8%) | 13 (22.0%) | 18 (38.3%) | 19 (35.2%) |  |  |
|  | Southwest | 12 (12.0%) | 14 (12.4%) | 6 (11.3%) | 10 (16.9%) | 6 (12.8%) | 4 (7.4%) |  |  |
|  | West | 14 (14.0%) | 27 (23.9%) | 5 (9.4%) | 15 (25.4%) | 9 (19.1%) | 12 (22.2%) |  |  |
| Level of Educational Attainment | Bachelor’s degree or higher | 58 (58.0%) | 50 (44.2%) | 33 (62.3%) | 24 (40.7%) | 25 (53.2%) | 26 (48.1%) | *P* = .095 | *P* = .04 |

Table S1D. Participant characteristics of total cohort - MTurk and Qualtrics Panels. P-values are differences between MTurk and Qualtrics panels cohorts within the Form and KIT sample populations.

|  |  |  |  |  | Confidence Interval | |
| --- | --- | --- | --- | --- | --- | --- |
|  | Estimate | Std. Error | t value | *P*-value | 2.5% | 97.5% |
| (Intercept) | 64.986 | 3.234 | 20.095 | <.001 ^a^ | 58.236 | 69.775 |
| Responses per minute | 1.130 | 0.492 | 2.298 | .021 ^b^ | 0.160 | 1.914 |
| UX Comments Likes length | 0.033 | 0.028 | 1.171 | .171 | -0.015 | 0.085 |
| UX comments Dislikes length | -0.033 | 0.025 | -1.339 | .348 | -0.066 | 0.023 |
| Duration | -0.197 | 0.129 | -1.535 | .233 | -0.369 | 0.090 |
| Intervention - KIT | 17.371 | 1.773 | 9.796 | <.001 ^a^ | 14.340 | 20.66820.870 |
| Cohort -Qualtrics Panels | -9.534 | 2.060 | -4.628 | <.001 ^a^ | -12.563 | -5.211 |

Table S2D. Multiple linear regression – SUS scores vs engagement characteristics. RSE: 12.31 on 192 degrees of freedom. Multiple R^2^: 0.461, Adjusted R^2^: 0.444. F-statistic: 27.32 on 6 and 192 DF. *P*-value: <.001 designated as ^a^, <.05 designated as ^b^

| **Chatbot Priority Features by Intervention** | **Form** | | | **KIT** | | | ***P*-value** |
| --- | --- | --- | --- | --- | --- | --- | --- |
| Rating | Gamification | Media elements | Personalization | Gamification | Media elements | Personalization | *P* = .381 |
| High priority | 26 (24.1%) | 20 (18.5%) | 50 (46.3%) | 22 (22.7%) | 15 (15.5%) | 64 (66.0%) |  |
| Medium priority | 34 (31.5%) | 43 (39.8%) | 46 (42.6%) | 32 (33.0%) | 43 (44.3%) | 28 (28.9%) |  |
| Low priority | 48 (44.4%) | 45 (41.7%) | 12 (11.1%) | 43 (44.3%) | 39 (40.2%) | 5 (5.2%) |  |

Table S3D. Chatbot priority features results between form and KIT participants
